# Connective auxin transport contributes to strigolactone-mediated shoot branching control independent of the transcription factor *BRC1*

**DOI:** 10.1371/journal.pgen.1008023

**Published:** 2019-03-13

**Authors:** Martin van Rongen, Tom Bennett, Fabrizio Ticchiarelli, Ottoline Leyser

**Affiliations:** Sainsbury Laboratory, University of Cambridge, Cambridge, United Kingdom; "USDA-ARS Pacific West Area", UNITED STATES

## Abstract

The shoot systems of plants are built by the action of the primary shoot apical meristem, established during embryogenesis. In the axil of each leaf produced by the primary meristem, secondary axillary shoot apical meristems are established. The dynamic regulation of the activity of these axillary meristems gives shoot systems their extraordinary plasticity of form. The ability of plants to activate or repress these axillary meristems appropriately requires communication between meristems that is environmentally sensitive. The transport network of the plant hormone auxin has long been implicated as a central player in this tuneable communication system, with other systemically mobile hormones, such as strigolactone and cytokinin, acting in part by modulating auxin transport. Until recently, the polar auxin transport stream, which provides a high conductance auxin transport route down stems dominated by the auxin export protein PIN-FORMED1 (PIN1), has been the focus for understanding long range auxin transport in the shoot. However, recently additional auxin exporters with important roles in the shoot have been identified, including PIN3, PIN4 and PIN7. These proteins contribute to a wider less polar stem auxin transport regime, which we have termed connective auxin transport (CAT), because of its role in communication across the shoot system. Here we present a genetic analysis of the role of CAT in shoot branching. We demonstrate that in Arabidopsis, CAT plays an important role in strigolactone-mediated shoot branching control, with the triple *pin3pin4pin7* mutant able to suppress partially the highly branched phenotype of strigolactone deficient mutants. In contrast, the branchy phenotype of mutants lacking the axillary meristem-expressed transcription factor, BRANCHED1 (BRC1) is unaffected by *pin3pin4pin7*. We further demonstrate that mutation in the ABCB19 auxin export protein, which like PIN3 PIN4 and PIN7 is widely expressed in stems, has very different effects, implicating ABCB19 in auxin loading at axillary bud apices.

## Author summary

The plant shoot system can be considered as a population of communicating growing tips, each driven by a shoot apical meristem. Communication between these meristems acts to mediate decisions about which meristems should be active and which dormant, depending on local and systemic environmental information, and hence how branchy a plant becomes. Here we analyse the role of transporters for the plant hormone auxin in this communication network and its ability to make meristem activity decisions. Previous work in this area has focussed on the auxin export protein PIN-FORMED1 (PIN1), which has a relatively narrow expression pattern in stems and is important for high conductance transport of auxin down toward the root. Here we analyse the unique contributions of more widely expressed auxin exporters, namely PIN3 PIN4 and PIN7, as well as ABCB19. We demonstrate that they have distinct roles in regulating axillary meristem activity. PIN3 PIN4 and PIN7 are likely important for communication between the axillary meristems and the main stem PIN1-dominated polar auxin transport stream. In contrast, our results suggest a role for ABCB19 in auxin loading at axillary shoot apices.

## Introduction

The co-ordinated regulation of plant growth requires effective communication across the plant body, prioritising growth where it is needed. A good example of this is the degree of shoot branching. The primary shoot axis of a plant is built by the activity of the shoot apical meristem, established during embryogenesis. The meristem produces stem underneath it and leaves at its flanks, and in the axil of each leaf, a new shoot apical meristem is established. These axillary meristems can become dormant buds after producing a few unexpanded leaves, or they can activate to produce a new shoot axis.

It has been known for many decades that shoot meristems are able to influence each other’s growth, a phenomenon that is particularly clear in apical dominance. Here, growth of the primary shoot prevents outgrowth of subtending axillary buds, and decapitation of the primary shoot releases axillary bud outgrowth. Apical dominance is mediated at least in part by the plant hormone auxin. Application of auxin to the decapitated stump of the primary shoot is able to restore axillary bud inhibition, even in axillary buds located far from the shoot tip [1], suggesting long-range auxin-mediated communication between shoot apices. Consistent with this idea, the young expanding leaves of growing shoots are known to be major sites for auxin biosynthesis [2] and this auxin is exported into the stem and transported towards the root in the polar auxin transport stream (PATS) (reviewed in [3]). Radiolabelled auxin applied apically is transported through the PATS to the root and can be detected in vascular-associated tissues in the stem, particularly the xylem parenchyma and the cambium. In Arabidopsis, these tissues are the sites of highly polar accumulation of the PIN1 auxin efflux carrier [4]. Mutation in PIN1 strongly reduces stem auxin transport, demonstrating an important role for PIN1 in the PATS [5].

Although it is clear that auxin moving in the PATS can inhibit the activity of axillary buds, the auxin does not enter the buds but rather acts indirectly [6, 7]. One mechanism by which auxin in the main stem could indirectly regulate bud activity is by reducing the ability of axillary buds to establish their own PATS out into the main stem. Establishment of high conductance auxin transport and PIN polarisation between the bud and the stem correlates with bud activation [8–12]. It likely proceeds by auxin transport canalisation, which is a process whereby an initial broad flow of auxin between an auxin source and an auxin sink becomes increasingly narrowed, upregulated, and polarised in the direction of the flow, generating files of cells that transport auxin between the source and the sink [13]. If sustained bud activation requires canalised transport of auxin between the bud and the stem PATS then it will depend on the relative bud auxin source strength and stem auxin sink strength, as well as the degree of positive feedback between auxin flux and auxin transporter accumulation and polarisation. Under these circumstances, auxin moving in the main stem can inhibit bud activity indirectly by reducing stem auxin sink strength. In this way all shoot apices compete to export their auxin into a common sink- the PATS in the main stem.

This model has previously been considered in the context of the behaviour of PIN1. However, recent work has shown that the stem auxin transport network is more complex than the unitary highly polar activity characteristic of the PATS [14–16]. Rather, current evidence suggest that stem auxin transport is multimodal with high-conductance, highly polar transport, as in the PATS, acting in concert with a more widespread low-conductance, less polar auxin transport activity, which we termed connective auxin transport (CAT) [14–16]. The PIN3, PIN4 and PIN7 auxin transporters are major contributors to CAT and play an important role in communication across tissues, since *pin3pin4pin7* mutant shoot apices show a reduced ability to communicate with each other [14]. In addition to PINs, the wide tissue distribution of ABCB family auxin transporters and their demonstrated contribution to bulk stem auxin transport suggests that they may also contribute to CAT [14, 17].

Communication between apices is also highly dependent on another class of plant hormones, strigolactones. Plants with mutations in the strigolactone pathway are highly branched (reviewed in [18]). Strigolactones are a class of carotenoid-derived compounds, which signal by binding to the D14 receptor triggering MAX2-dependent degradation of a small family of HSP101-like proteins, in Arabidopsis SMXL6, SMXL7 and SMXL8 [19, 20]. Strigolactones appear to influence branching in two distinct ways. Firstly, they modulate auxin transport by triggering endocytosis of PIN1 from the plasma membrane [21, 22]. This is predicted to dampen the feedback between auxin flux and auxin transport, making it more difficult for buds to establish canalised auxin flow into the main stem, which is hypothesised to reduce branching [12]. In effect, through this mode of action strigolactone enhances the level of competition between buds. This increase in competition is most evident when stem segments bearing two buds are supplied basally with exogenous strigolactone. Rather than inhibiting the growth of both buds, strigolactone focuses growth into a single branch [21].

The second mode of action for strigolactones in reducing branching is through local up-regulation of the expression of the *TEOSINTE BRANCHED1* (*TB1*) class of TCP transcription factors in buds. In maize, overexpression of *TB1* inhibits the activity of axillary buds, leading to plants that are less branched than their wild relative teosinte [23]. *TB1-*related genes have been identified in multiple species, including *FINE CULM1* (*FC1*) in rice and *BRANCHED1* (*BRC1*) in Arabidopsis, tomato, potato and pea [24–31]. Loss of *BRC1* in Arabidopsis leads to increased branching, reduced bud-bud communication in 2-node explants, and a reduced response of branches to strigolactone [24, 28, 32, 33]. Strigolactone can up-regulate the transcription of *BRC1*, independent of new protein synthesis [27]. Furthermore, mutants deficient in strigolactone synthesis or perception have very low levels of bud *BRC1* transcripts, and Arabidopsis mutants lacking SMXL6, SMXL7 and SMXL8 proteins have constitutively high bud *BRC1* transcript levels [19, 26, 27]. Interestingly, *BRC1* expression is high even in active buds of *smxl6 smxl7 smxl8* triple mutants, demonstrating that high *BRC1* expression is not sufficient for bud inhibition [33]. Furthermore, many buds in *brc1* mutant plants can remain dormant, demonstrating that high *BRC1* is not necessary for bud inhibition [33].

These data demonstrate that loss of strigolactone signalling, loss of *BRC1* and loss of the CAT PINs, PIN3 PIN4 and PIN7 all reduce bud-bud communication and/or competition. This raises the interesting question of the relationship between these factors, and more generally of the role of CAT in bud activation. Here we analyse the effects of CAT mutants in order to address these questions. We conclude that CAT is important for bud activation and that strigolactone-mediated shoot branching control is strongly affected by CAT, independent of effects on *BRC1*. We further demonstrate that ABCB19 action is distinct from that of PIN3 PIN4 and PIN7 and is involved in both strigolactone and *BRC1*-dependent bud regulation.

## Results

### CAT contributes to strigolactone-mediated branching

**Fig 1:**
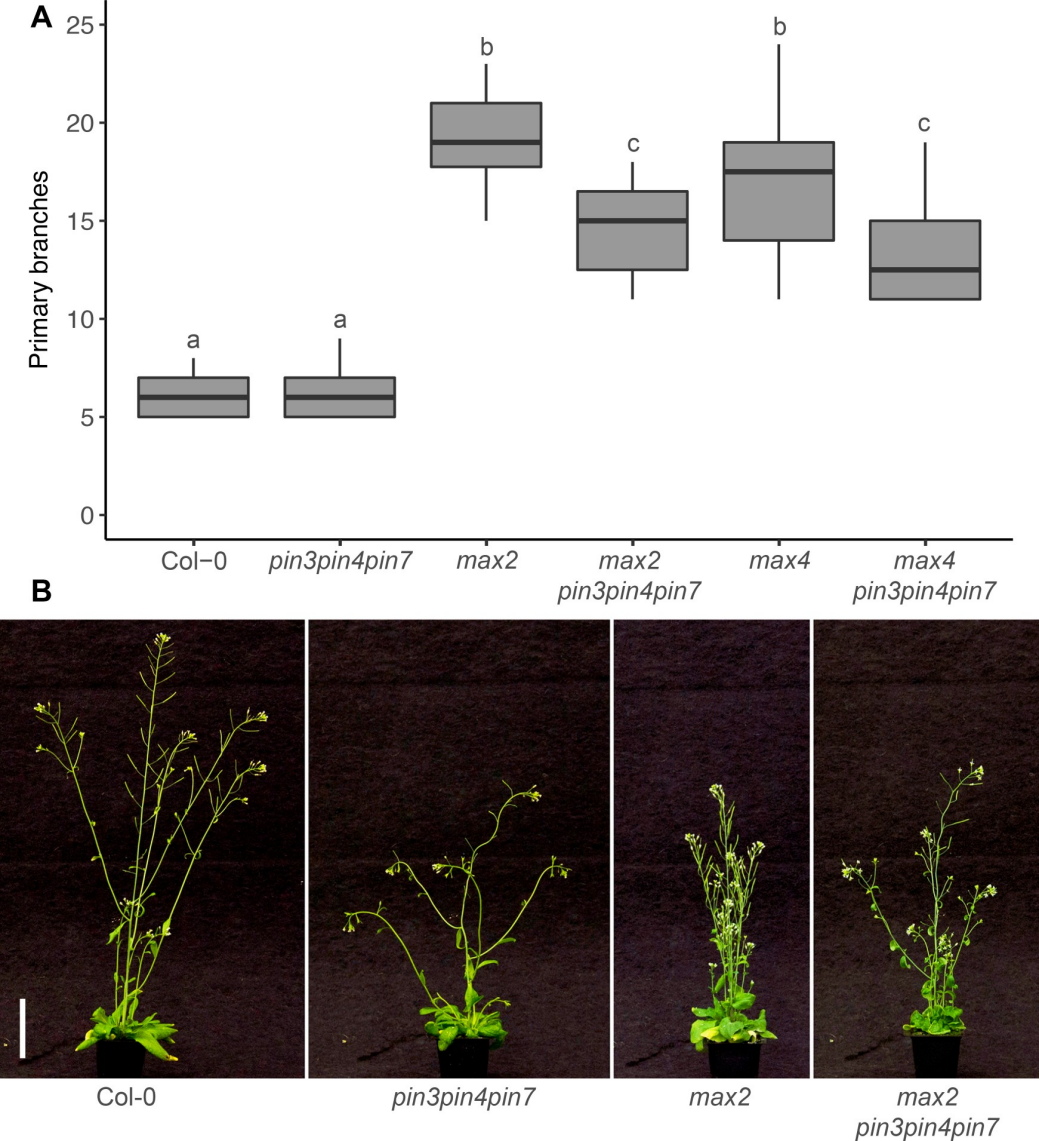
PIN347 are required for full bud activation in strigolactone mutants. (A) Number of primary branches at terminal flowering for the genotypes indicated. (B) Representative plants of the genotypes in (A) at 6 weeks post-germination. Bar = 50 mm. In (A) The boxes span the first to third quartile and the line represents the median. The whiskers indicate the variability outside the upper and lower quartiles. Tukey’s HSD test was carried out after obtaining the least-square means for a linear model fitting the data and different letters indicate statistically significant differences at *p* < 0.05, *n* = 12–20.

To assess the role of the CAT PINs, PIN3 PIN4 and PIN7, in strigolactone-mediated shoot branching control, we generated quadruple mutants between these PINs and the strigolactone biosynthetic mutant, *max4-5*, hereafter *max4*, or the strigolactone signalling mutant *max2-1*, hereafter *max2*. Consistent with earlier findings, branching in the *pin3-3pin4-3pin7-1* triple mutant, hereafter *pin347*, under standard long day growth conditions was not markedly different from wild type (Fig 1; [14]), whereas *max2* and *max4* mutants were highly branched (Fig 1; [34, 35]). Loss of PIN347 in the *max2* and *max4* mutant backgrounds significantly reduced shoot branching (Fig 1), demonstrating that these PINs are required for full bud activation under strigolactone deficiency. The single and double *PIN* mutant combinations we tested had little effect on branching in the *max2* background (S1A Fig).

In addition to increased branching, strigolactone mutants display a syndrome of characteristic shoot phenotypes, including more acute branch angles, shorter stature and thinner stems than wild type [34, 36]. Since *pin347* mutants display more obtuse branch angles than wild type [14], we measured branch angle in *max2pin347* and *max4pin347* plants. In both these mutant backgrounds branch angle was restored to wild-type (S1B Fig). Because the *maxpin347* mutants also appeared taller than the strigolactone mutants, we quantified plant height and found that that loss of PIN347 was able to restore partially the short stature of *max* mutants (S1C Fig). No effect was observed on stem diameter (S1D Fig).

*MAX2* is known to act not only in strigolactone signalling, with perception mediated by the D14 protein, but also in KAI2-mediated signalling of karrikin and its presumed endogenous karrikin-like ligand [19]. We have previously shown that KAI2 is not involved in the control of shoot branching [36]. Nonetheless, to confirm that the *max2* phenotypes are indeed due to lack of strigolactone signalling, we used the *d14-1* strigolactone receptor mutant [37], hereafter *d14*, and introduced into it the *pin347* mutations. Consistent with the *max2pin347* phenotypes, branching was reduced in *d14pin347* mutants (S1E Fig), branch angles were less obtuse (S1F Fig), plant height was increased (S1G Fig) and stem diameter was unchanged (S1H Fig).

### CAT contributes to increased stem auxin transport in strigolactone mutants

**Fig 2:**
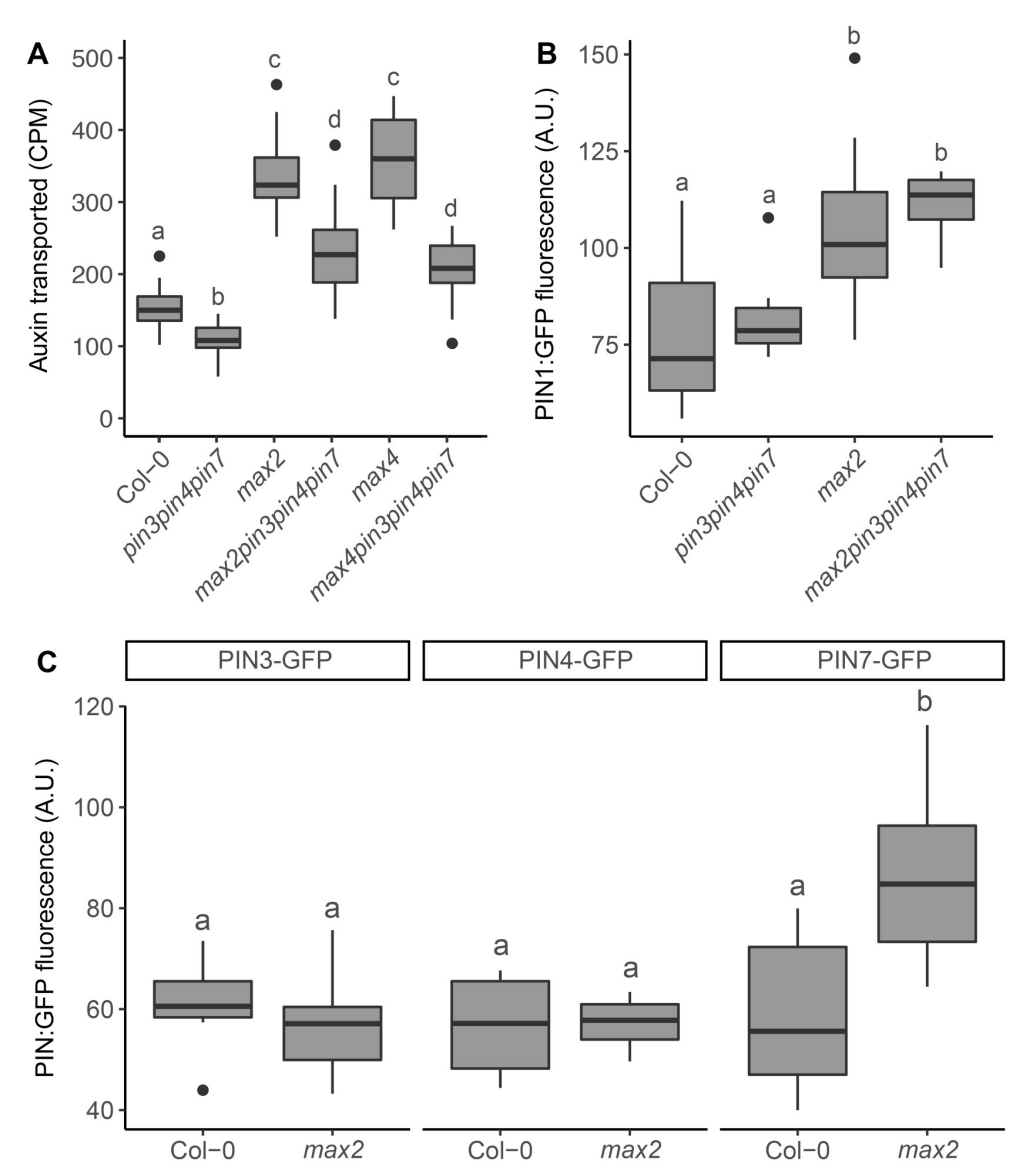
Loss of PIN347 reduces stem auxin transport in strigolactone mutants without affecting PIN1. (A) Bulk stem auxin transport in basal inflorescence stem internodes of 6-week old plants of the genotypes indicated. Transport was determined as basal accumulation of radiolabeled auxin, quantified as counts per minute (CPM) after 6-hours incubation in 1 μM ^14^C-IAA. (B) Quantification of PIN1-GFP in arbitrary units (A.U) at the basal plasma membrane of xylem parenchyma cells in longitudinal hand sections through basal inflorescence internodes of 6-week old plants of the genotypes indicated, homozygous for *PIN1*::*PIN1-GFP*. (C) Quantification of PIN3-GFP, PIN4-GFP and PIN7-GFP in arbitrary units (A.U.) at the basal plasma membrane of xylem parenchyma cells in longitudinal hand sections through basal inflorescence internodes of 6-week old *max2* plants. The boxes span the first to third quartile and the line represents the median. The whiskers indicate the variability outside the upper and lower quartiles and outliers are indicated by individual points. Tukey’s HSD test were carried out after obtaining the least-square means for a linear model fitting the data and different letters indicate statistically significant differences at *p* < 0.05. For (A), *n* = 19–24. For B and C statistical analyses were carried our using the mean of 5 membranes from 4–8 plants per line.

The increased branching phenotype of the *max* mutants can at least in part be attributed to reduced PIN1 removal from the plasma membrane, which results not only in increased branching, but also in increased PIN1 accumulation and bulk auxin transport in the stem [21, 22, 38]. To determine whether loss of PIN347 affects *max* stem auxin transport, we measured bulk auxin movement in relevant genotypes using our standard auxin transport assay. Consistent with previous findings, stem auxin transport is reduced in *pin347* mutants and increased in *max* mutants (Fig 2A; [14, 38]). Loss of PIN347 in the *max2* and *max4* mutant backgrounds reduced bulk stem auxin transport more strongly than in the wild-type background (Fig 2A).

To determine whether the reduction of stem auxin transport in *max pin347* mutants is associated with reduced accumulation of PIN1, we crossed a *PIN1*::*PIN1-GFP* reporter into the *max2pin347* mutant background and quantified GFP on the basal plasma membrane of stem xylem parenchyma cells. Consistent with earlier reports, *max2* stems displayed high levels of PIN1 (Fig 2B; [22]). Loss of PIN347 had no effect on PIN1 accumulation in either wild-type or *max2* mutant backgrounds (Fig 2B).

To assess the effect of *max2* mutation on the accumulation of PIN3, PIN4 and PIN7, we crossed *PIN3*::*PIN3-GFP*, *PIN4*::*PIN4-GFP* and *PIN7*::*PIN7-GFP* reporter constructs into the *max2* mutant background and quantified GFP on the basal plasma membrane of stem xylem parenchyma cells. No differences in PIN3 and PIN4 accumulation could be detected between wild type and *max2*, whereas PIN7 accumulation was increased in the *max2* mutant background (Fig 2C).

Together these data suggest that CAT PINs contribute to auxin transport and shoot branching in strigolactone mutants, and while PIN7 accumulation might be regulated by strigolactone, this alone is insufficient to account for the effects of *pin347* mutation on branch suppression in *max* backgrounds.

### CAT contributes to strigolactone-mediated bud-bud competition

**Fig 3:**
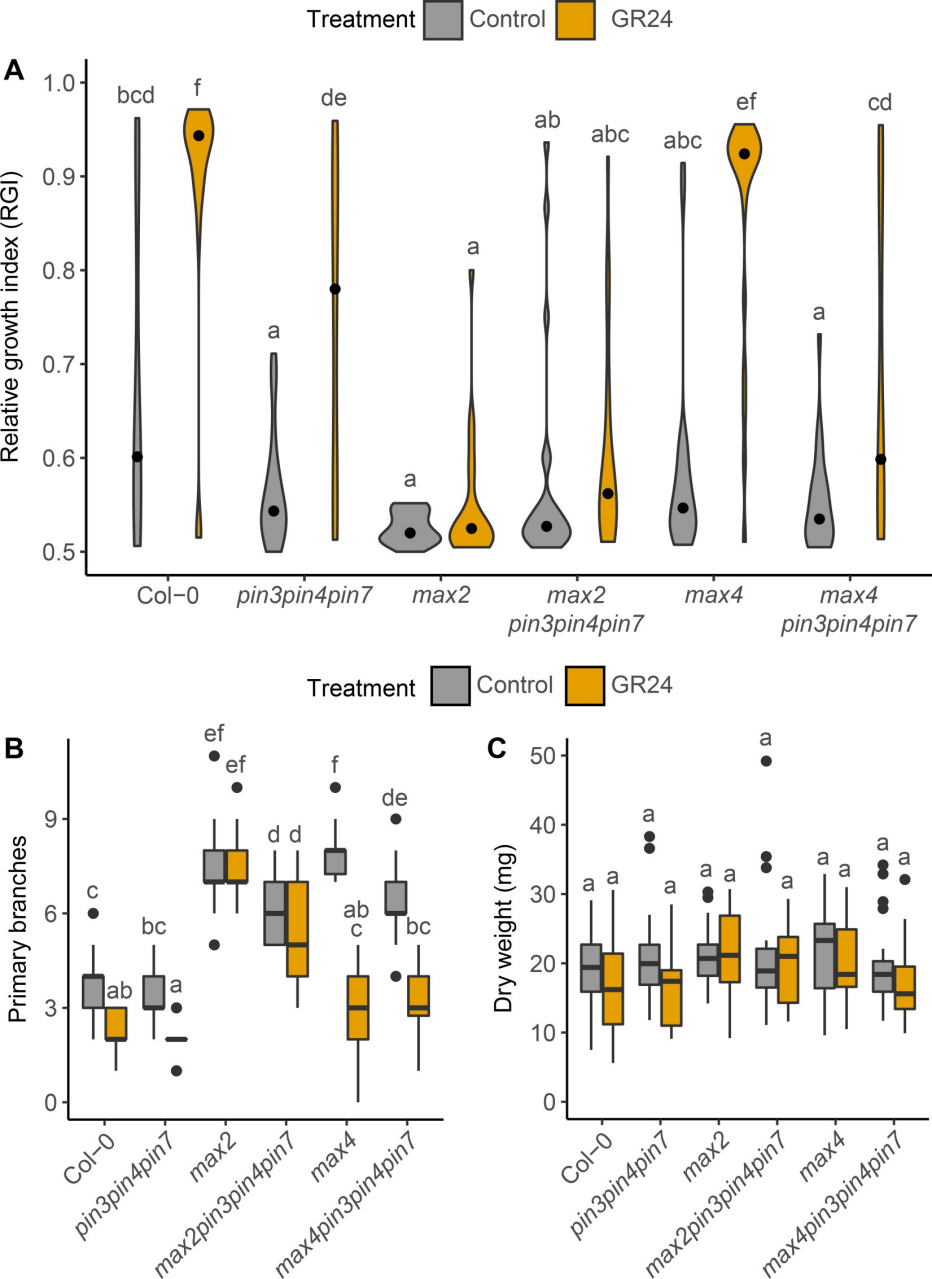
PIN347 contribute to strigolactone-mediated bud-bud competition. (A) Violin plots of the relative growth index (RGI) of 2-node explants of the genotypes indicated 10 days post decapitation, with (orange) or without (grey) 5 μM GR24 supplied basally. The RGI is the proportion of branch length in the longest branch. Black dots indicate the median value and the area of each plot represents the probability distribution of the values, *n* = 20–23. (B) Primary branch number of plants of the genotypes indicated grown axenically on ATS with (orange) or without (grey) 5 μM GR24 in long days, scored after 8 weeks, *n* = 14–21. (C) Dry weight of the plants represented in B. For B and C the boxes span the first to third quartile and the line represents the median. The whiskers indicate the variability outside the upper and lower quartiles. For A-C Tukey’s HSD tests were carried out after obtaining the least-square means for a linear model fitting the data and different letters indicate statistically significant results at *p* < 0.05.

Competition between buds can be assayed using isolated stem explants bearing two nodes, and hence two axillary buds [39]. Typically, either one bud or both buds activate, and the degree of competition between buds is reflected in the proportion of these two outcomes. One measure of this is the relative growth index (RGI), which is the length of the longest branch divided by the sum of the lengths of both branches [39]. Bud-bud competition in this assay is strongly reduced in strigolactone mutants as well as in the *pin347* mutant background [14, 39], whereas strigolactone supplied basally through the stem increases competition between buds [21]. To determine whether *pin347* buds on two-node explants are still responsive to exogenous strigolactone, we determined the RGI of explants supplied basally with 5 μM *rac-*GR24, a synthetic strigolactone, or solvent control (Fig 3A). As previously observed, *pin347* two-node explants without treatment display decreased bud-bud competition, with both buds often activating, resulting in a low RGI compared to wild type (Fig 3A; [14]). Addition of basal GR24 increased bud-bud competition, as indicated by the increased RGI, but not to wild-type levels (Fig 3A). Consistent with these results, loss of PIN347 in the *max4* background led to a partial response to basal GR24, whereas GR24 restored *max4* bud-bud competition to levels comparable to wild type (Fig 3A). As expected, RGI was not significantly affected by basal GR24 supply in the strigolactone-insensitive *max2* and *max2pin347* mutants (Fig 3A).

To determine whether the partial response of *pin347* to exogenous strigolactone application is also observed in intact plants, we grew plants under axenic conditions in jars, with 5 μM *rac*-GR24 added to the growth medium, or a solvent control. Branching was assessed 8 weeks after sowing, at which point all plants had reached terminal flowering. Both wild type and *pin347* plants responded to strigolactone treatment by reducing branching (Fig 3B). As for soil-grown plants, the loss of PIN347 reduced branching in both the *max2* and *max4* mutant backgrounds (Fig 3B). Both *max4* and *max4pin347* plants responded to basal application of GR24 with branching reduced to levels comparable to wild type GR24 treated plants (Fig 3B). As expected, addition of basal GR24 had no discernible effect in *max2* and *max2pin347* mutant plants (Fig 3B).

Growing plants on GR24 can affect overall plant vigour, which is reflected in total dry weight [22]. To determine whether differences in branching could be attributed to changes in overall vigour, the dry weight of the plants used to assess branching was determined. No difference in dry weight could be detected between any of the genotypes or treatments (Fig 3C).

Together these data suggest that PIN347 contribute to the ability of buds to respond to strigolactone, but this can only be detected in the more sensitive bud-bud competition assay.

### ABCB19 contributes to shoot branching

**Fig 4:**
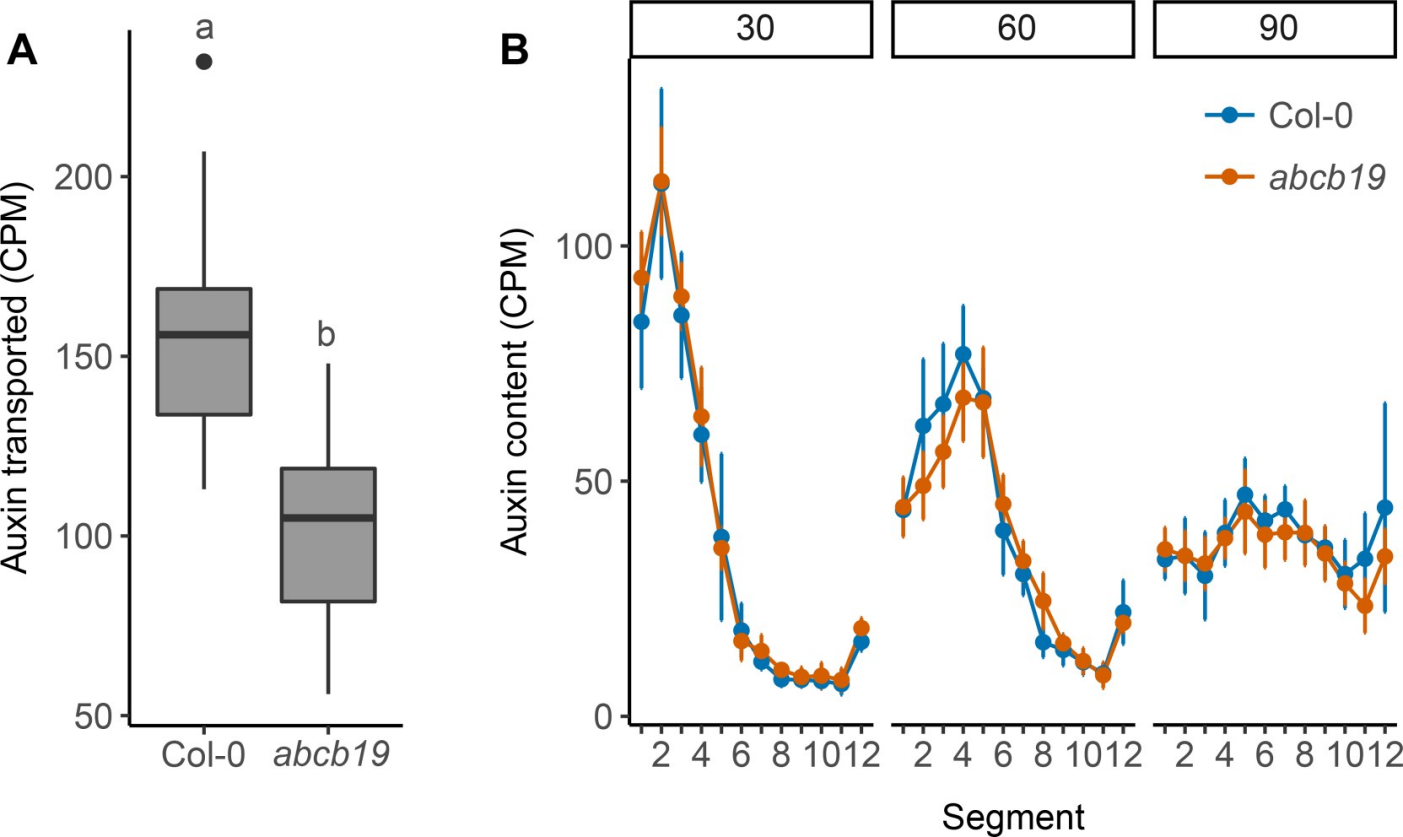
ABCB19 contributes to stem auxin transport. (A) Bulk stem auxin transport through 15 mm basal inflorescence internodes of 6-week old Col-0 and *abcb19* plants. Transport was determined as basal accumulation of radiolabeled auxin, quantified as counts per minute (CPM) in the basal 5 mm of stem after 18 hours of incubation of the apical end 1 μM ^14^C-IAA. (B) Progression of a 10-minute pulse of 5 μM ^14^C-IAA applied to the apical end of 24 mm long basal inflorescence internodes of 6-week old Col-0 and *abcb19* plants assessed at 30, 60 and 90 minutes after application of the pulse (left to right), shown as mean CPM in 2 mm sections of the stems. For A the boxes span the first to third quartile and the line represents the median. The whiskers indicate the variability outside the upper and lower quartiles and outliers are indicated by individual points. Tukey’s HSD test was carried out after obtaining the least-square means for a linear model fitting the data and different letters indicate statistically significant differences at *p* < 0.05, *n* = 24. For B the error bars represent the 95% confidence interval of the mean, *n* = 8 for each point. No significant differences were detected at any time point (unadjusted Wilcoxon rank-sum tests).

In addition to PIN proteins, ABCB auxin transporters also affect stem auxin transport [17]. Loss of two closely related members, ABCB1 and ABCB19, leads to severe growth defects and strongly reduced stem auxin transport [17]. Both ABCB1 and ABCB19 are expressed in a predominantly non-polar manner in a variety of tissues [40, 41], with ABCB19 non-polarly expressed across the stem [14]. Combined, these data suggest that ABCB1 and ABCB19 could contribute to CAT. However, we have previously been unable to detect consistent significant changes in bulk stem auxin transport in either single mutant, and the double mutant data are difficult to interpret owing to the severe morphological phenotypes [14]. To explore in more detail the role of ABCB19 in stem auxin transport in general, and CAT in particular, we extended our analysis of the effects of its mutation. Our standard bulk auxin transport assay is conducted over a 6-hour period [14]. To increase the sensitivity of this assay we assessed transport over an 18-hour period. This revealed a clear reduction in bulk auxin transport between the *abcb19-101* mutant, hereafter *abcb19*, and wild type (Fig 4A).

The concept of CAT was introduced to account for the kinetics of auxin transport in Arabidopsis stems. When a pulse of radiolabelled auxin is applied to the apical end of an Arabidopsis stem segment its progress down the stem can be followed by cutting the stem into small segments and counting their radiolabel content [14]. The auxin pulse rapidly spreads in a manner consistent with at least two modes of stem auxin transport with exchange between them, namely PATS, consisting of high capacity highly polar transport, and CAT consisting of less polar lower capacity transport. Evidence supporting a role for PIN347 in CAT and particularly in the exchange of auxin between CAT and the PATS is the bimodal progress of an auxin pulse down the stem of *pin347* mutants, with some auxin moving more slowly than wild type, presumably because it is trapped in CAT tissues, and some auxin moving faster, presumably because it is trapped in the PATS [14]. To assess the contribution of ABCB19 to this exchange, we followed the movement of a pulse of auxin transport down the stems of *abcb19* mutants. No differences from wild type were detected at any of the sampled time points, suggesting that ABCB19 is not required for efficient exchange of auxin between the PATS and surrounding CAT tissues (Fig 4B).

**Fig 5:**
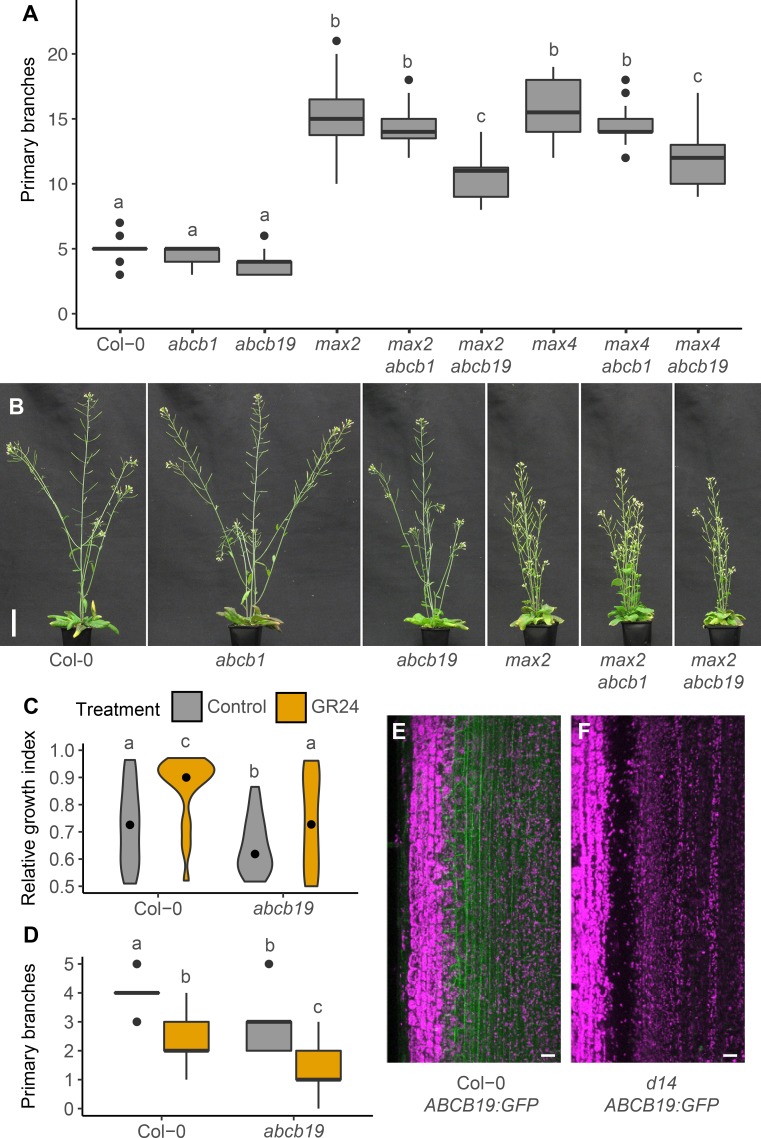
ABCB19 is required for full bud activation in strigolactone mutants. (A) Number of primary branches at terminal flowering for the genotypes indicated. The boxes span the first to third quartile and the line represents the median. The whiskers indicate the variability outside the upper and lower quartiles and outliers are indicated by individual points, *n* = 20–24. (B) Representative plants of the genotypes in A at 6 weeks post-germination. Bar = 50 mm. (C) Violin plot of the relative growth index (RGI) of 2-node explants 10 days post decapitation with (orange) or without (grey) 5 μM GR24 supplied basally. The RGI is the proportion of branch length in the longest branch. Black dots indicate the median value and the area of each plot represents the probability distribution of the values, *n* = 21–24. (D) Primary branch number of 8-week old plants grown axenically in long day conditions on ATS with (orange) or without (grey) 5 μM GR24. The boxes span the first to third quartile and the line represents the median. The whiskers indicate the variability outside the upper and lower quartiles, *n* = 20–21. (E) Representative maximum projection image showing expression of *ABCB19*::*ABCB19-GFP* in longitudinal hand sections through basal inflorescence internodes of 6-week old wild-type stems, *n* = 22. (F) Representative maximum projection image showing expression of *ABCB19*::*ABCB19-GFP* in longitudinal hand sections through basal inflorescence internodes of 6-week old *d14* mutant stems, *n* = 16. For A, C and D Tukey’s HSD tests were carried out after obtaining the least-square means for a linear model fitting the data and different letters indicate statistically significant differences at *p* < 0.05. For E and F the scale bar is 50 μm.

We have previously shown that mutation in *ABCB1* and *ABCB19* have no effect on shoot branching in our standard soil-grown conditions. To assess their interaction with strigolactone signalling, we introduced the *abcb1-100*, hereafter *abcb1*, and *abcb19* mutations into the *max2* and *max4* backgrounds and determined the degree of branching. Whereas loss of ABCB1 did not affect branching in either *max* mutant, loss of ABCB19 reduced the levels of shoot branching in both (Fig 5A and 5B). We also determined whether other strigolactone-related shoot phenotypes were affected and found that loss of ABCB19 resulted in a small increase in branch angle in *max2* and *max4* mutant plants, whereas loss of ABCB1 had no effect (S2A Fig). Plant height and stem diameter were unaffected by *abcb1* and *abcb19* in any of the genetic backgrounds tested (S2B and S2C Fig).

Because loss of ABCB19 in strigolactone mutant backgrounds reduces branching in intact plants, we wondered whether *abcb19* mutants were strigolactone resistant. To test this, we assayed the strigolactone response of *abcb19* mutants in bud-bud competition assays and in intact plants grown axenically in jars. We found that untreated *abcb19* two-node explants displayed reduced bud-bud competition compared to wild type and that treatment with basal GR24 was only partially able to rescue this phenotype (Fig 5C). Branching in jar-grown *abcb19* mutants was reduced by GR24 addition, with branching levels lower than wild type for both untreated and GR24-treated plants (Fig 5D).

To determine whether ABCB19 protein accumulation is altered in strigolactone deficient mutants, we introduced an *ABCB19*::*ABCB19-GFP* reporter construct into *d14* and assessed protein accumulation at the xylem parenchyma. Consistent with previous data [14] ABCB19 is detected in the majority of the cells in the stem in wild type and displays a predominantly non-polar localisation (Fig 5E). In contrast, ABCB19 could not be detected in any stem tissues in the *d14* mutant background (Fig 5F).

Together these data suggest that ABCB19 is involved in strigolactone-mediated shoot branching control, despite its limited effect on stem auxin transport.

### ABCB19 and PIN347 have additive effects on bud activation kinetics

**Fig 6:**
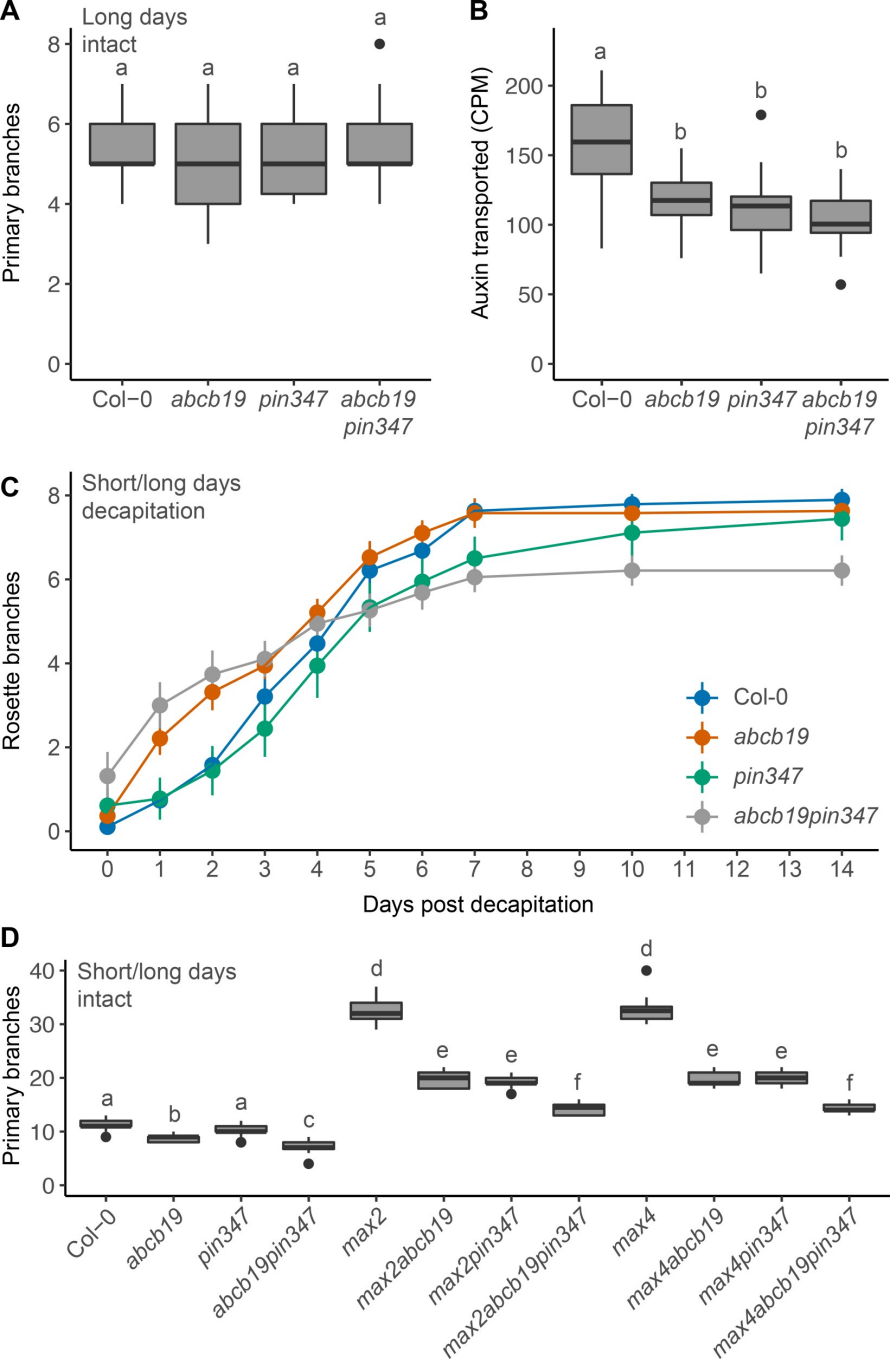
PIN347 and ABCB19 have contrasting effects on bud activation kinetics. (A) Primary branch number at terminal flowering of plants of the genotypes indicated grown under long day growth conditions. *n* = 18–24. (B) Bulk stem auxin transport through 15 mm basal inflorescence internodes of 6-week old plants of the genotypes indicated. Transport was determined as basal accumulation of radiolabeled auxin, quantified as counts per minute (CPM) in the basal 5 mm of stem after 18 hours of incubation of the apical end 1 μM ^14^C-IAA, *n* = 24. (C) Mean number of active rosette branches over time following decapitation at day 0 for Col-0 (blue), *abcb19* (orange), *pin347* (green) and *abcb19pin347* (grey) plants. Plants were grown under short day conditions for 4 weeks, shifted to long days to induce flowering and decapitated when the inflorescences reached 10 cm. The number of active rosette branches, defined as longer than 5 mm were counted daily. Error bars represent the 95% confidence interval of the mean. Non-overlapping error bars indicate statistical differences compared to wild type, verified using non-parametric tests comparing wild type and each mutant with a threshold of *p* < 0.05, with Holm-Bonferroni adjustment, *n* = 18–19. (D) Number of primary branches at terminal flowering for plants of the genotypes indicated grown under short day conditions for 4 weeks and then shifted to long days, *n* = 20–24. For A, B and D the boxes span the first to third quartile and the line represents the median. The whiskers indicate the variability outside the upper and lower quartiles and outliers are indicated by individual points. Tukey’s HSD tests were carried out after obtaining the least-square means for a linear model fitting the data and different letters indicate statistically significant differences at *p* < 0.05. For C, Holm-Bonferroni corrected Wilcoxon rank-sum tests were used to test between genotypes and time points as indicated in the text.

These results reveal some similarities between the shoot branching phenotypes of the *abcb19* and *pin347* mutants, but significant differences in their effects on stem auxin transport dynamics. To investigate the relationship between these transporters in more detail, we created *abcb19pin347* quadruple mutants and measured bud outgrowth responses in various contexts. No clear effect on branching levels at maturity could be detected under standard long day conditions (Figs 6A and S3D). We also characterised several other shoot phenotypes in the quadruple mutant. Branch angles were significantly increased compared to wild type in *abcb19pin347* plants but were no different from *pin347* plants (S3A Fig). Plant height and stem diameter were significantly reduced in *abcb19pin347* plants compared to all the other genotypes assayed (S3B and S3C Fig).

We next measured bulk auxin transport through *abcb19pin347* stems over an 18-hour period (Fig 6B). Consistent with previous results (Figs 2A and 4A), *abcb19* and *pin347* stems displayed reduced stem auxin transport compared to wild type (Fig 6B). Auxin transport in *abcb19pin347* stems was no different from either *abcb19* or *pin347* (Fig 6B).

To assess bud activation in more detail, we followed bud activation over two weeks in a well-established decapitation assay [14, 42]. In this assay, wild-type plants activate buds at a rate of approximately one per day over a period of a week, after which no further buds activate (Fig 6C). The rate of bud activation gradually increases and then decreases over this week. The *pin347* triple mutant showed similar bud activation kinetics to wild type for the early part of the time course, but activation then slowed earlier than in the wild type, such that there were fewer activated branches than wild type at 7 days (*p* < 0.05, Holm-Bonferroni adjusted Wilcoxon rank-sum tests), but no significant difference by 10 days onwards (Fig 6C), consistent with previously observed trends [14]. In contrast, the *abcb19* mutant activated buds more rapidly than wild type over the first two days following decapitation (*p* < 0.05, Holm-Bonferroni adjusted Wilcoxon rank-sum tests) (Fig 6C), with activation rate decreasing over time such that branch numbers converged with wild type at day three. The *abcb19pin347* quadruple mutant showed an essentially additive phenotype, combining the rapid early activation rate of *abcb19* with early slowing, similar to *pin347*. Total branching was reduced compared to the other genotypes by the end of the experiment (Fig 6C).

To determine the effects of loss of both ABCB19 and PIN347 in strigolactone mutant backgrounds, we created *max abcb19pin347* quintuple mutants. Since the additive effects of *abcb19* and *pin347* are too small detect under standard long day growth conditions (Fig 6A), we assessed intact branching under conditions where plants were initially grown under short day conditions for four weeks and then shifted to long day growth conditions. Growth under short days prolongs the vegetative phase and increases the number of vegetative nodes, which typically all activate in *max* mutant backgrounds. Plants were scored at terminal flowering. As in long day conditions, *max abcb19* and *max pin347* mutants displayed reduced branching compared to *max* mutants alone (Fig 6D). Branching in *max abcb19pin347* mutants was further reduced compared to *max abcb19* and *max pin347* mutants (Fig 6D). Branching in *pin347* was not significantly different from wild type, as in long day conditions (Figs 1A and 6A). In contrast, branching in *abcb19* mutants was significantly decreased under these growth conditions, with a further reduction observed in the *abcb19pin347* quadruple mutant (Fig 6D). This differs from *abcb19* branching under long days, where no difference from wild type was detected (Figs 5A and 6A).

### *BRC1*-mediated branching is differentially dependent on PIN347 and ABCB19

The data presented above demonstrate a strong interaction between stem-expressed auxin transporters and strigolactone-mediated branching. In addition to modulating auxin transport, strigolactone affects branching by increasing *BRC1* transcript abundance [26, 27, 33]. *BRC1* transcript levels correlate strongly with bud inhibition and loss of *BRC1* results in increased branching [24, 28]. We have previously shown that *BRC1* mutants have wild-type bulk auxin transport levels and PIN1 accumulation in the xylem parenchyma, indicating that its effects on shoot branching are PIN1-independent [36]. To determine the relationship between *BRC1* and CAT, we introduced the *pin347* and *abcb19* mutations into the *brc1-2brc2-1*, hereafter *brc1brc2*, mutant background.

**Fig 7:**
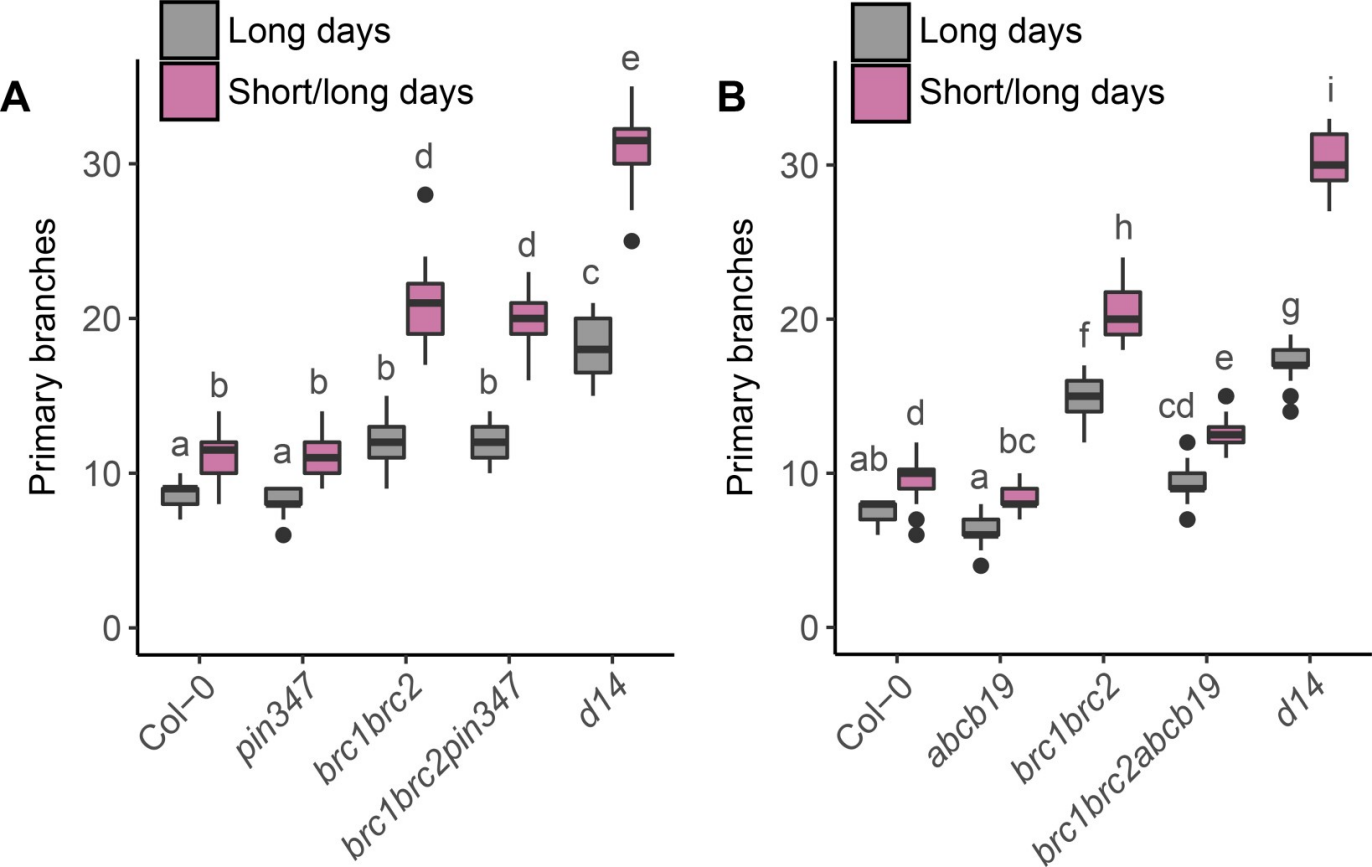
Shoot branching in *brc1brc2* is independent of PIN347 but partially dependent on ABCB19. (A) and (B) Primary branch number at terminal flowering in plants of the genotypes indicated grown continuously under long day conditions (grey) or under short day conditions for four weeks and then shifted to long day conditions (magenta), *n* = 20–24. The boxes span the first to third quartile and the line represents the median. The whiskers indicate the variability outside the upper and lower quartiles. Tukey’s HSD tests were carried out after obtaining the least-square means for a linear model fitting the data and different letters indicate statistically significant differences at *p* < 0.05.

Mutants lacking BRC1, and to a lesser extent the closely related BRC2, show increased branching compared to wild type [24, 28]. In long-day conditions almost all nodes produce a branch, but under short-day conditions, where many more nodes are formed, many nodes carry inactive buds [33]. In contrast, strigolactone mutants such as the *d14* strigolactone receptor mutant, display a higher degree of branching than *brc1brc2* mutants, and this is particularly clear under conditions where many nodes are produced (Fig 7; [24, 33, 36]). When *brc1brc2pin347* quintuple mutants were grown in such conditions, no differences in branching compared to *brc1brc2* were detected (Fig 7A). However, loss of ABCB19 significantly reduced branching in *brc1brc2* mutants in both short-day/long-day shift experiments and in standard long-day growth conditions (Fig 7B). Notably, branching in the *abcb19* single mutant grown under short day/long day conditions was reduced compared to wild type (Fig 7B), an effect consistent with previous data (Fig 6D). No effect of *abcb1* was detected on *brc1brc2* branching (S4 Fig).

**Fig 8:**
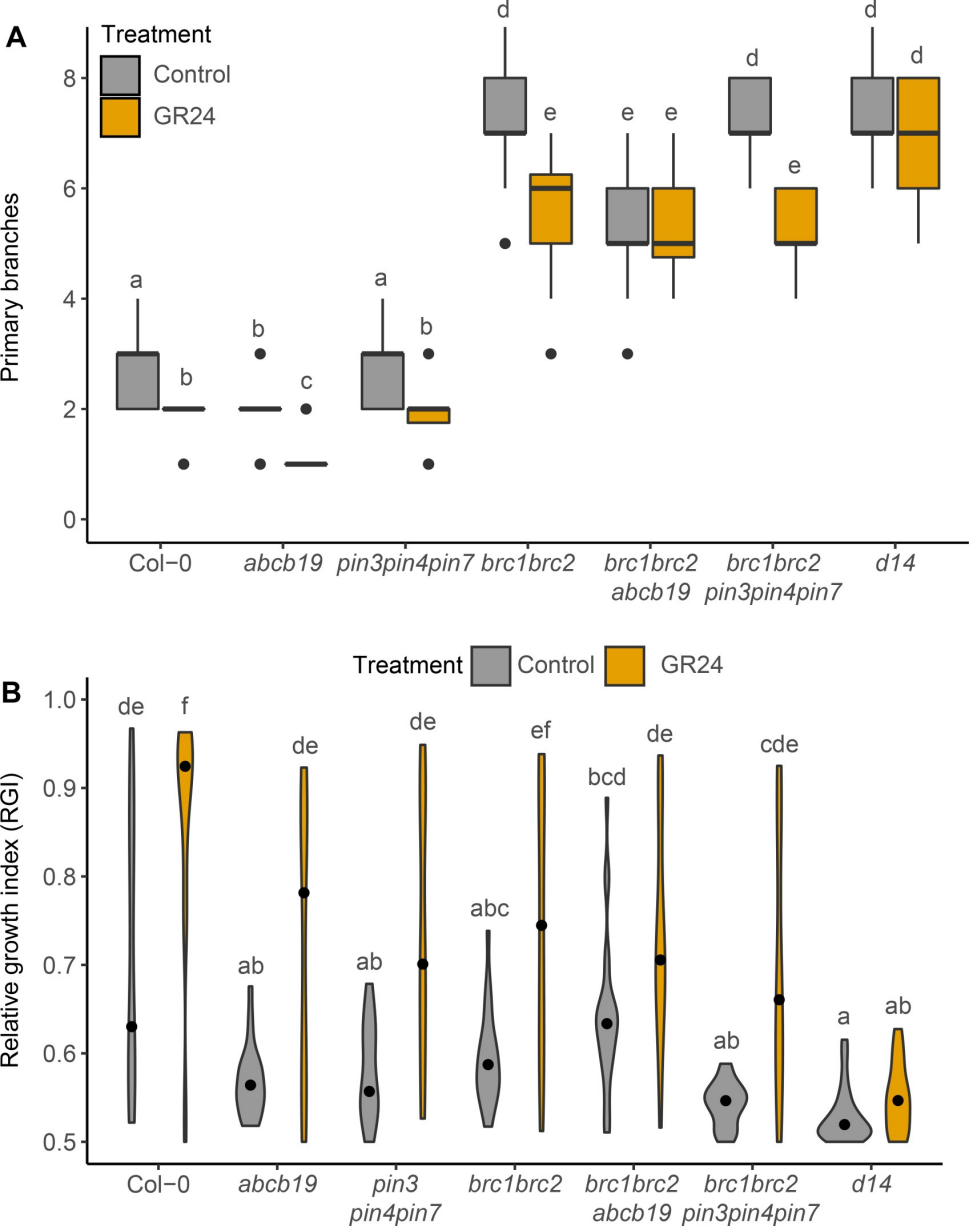
PIN347 and ABCB19 mutation have limited impact on strigolactone response in *brc1brc2* mutant branching. (A) Primary branch number of plants of the genotypes indicated grown axenically on ATS with (orange) or without (grey) 5 μM GR24 in long days, scored after 8 weeks, *n* = 24–28. The boxes span the first to third quartile and the line represents the median. The whiskers indicate the variability outside the upper and lower quartiles. (B) Violin plots of the relative growth index (RGI) of 2-node explants of the genotypes indicated 10 days post decapitation, with (orange) or without (grey) 5 μM GR24 supplied basally, The RGI is the proportion of branch length in the longest branch. Black dots indicate the median value and the area of each plot represents the probability distribution of the values, n = 20–24. For A and B Tukey’s HSD tests were carried out after obtaining the least-square means for a linear model fitting the data and different letters indicate statistically significant differences at *p* < 0.05.

We have previously shown that *brc1brc2* mutants show a reduced but significant response to strigolactone [33]. We assessed whether this responsiveness is retained in *brc1brc2* mutants lacking PIN347 or ABCB19. For whole plants grown axenically, the *brc1brc2* and *brc1brc2pin347* plants produced fewer branches on 5μM GR24 compared to their untreated controls. The *brc1brc2abcb19* plants showed no response to GR24 treatment (Fig 8A). To explore this further, we used a bud-bud competition assay. Consistent with our previous findings, bud-bud competition in *brc1brc2* 2-node explants was reduced compared to wild type (Fig 8B; [33]). There was no evidence for additivity between *brc1brc2* and either *pin347* or *abcb19* in this assay (Fig 8B). The RGI of *brc1brc2pin347* and *brc1brc2abcb19* mutants under GR24 treatment was not significantly different from that of the *brc1brc2* double mutant (Fig 8B).

## Discussion

Auxin transport plays a central role in shoot branching. This role has been largely attributed to the Polar Auxin Transport Stream (PATS), in which PIN1 plays a critical role [4, 5, 43]. Loss of PIN1 results in highly pleiotropic phenotypes, including failure to produce axillary meristems [5, 44], making its specific role hard to assess. Here we have established an important role for auxin transporters with wider and less polar accumulation, specifically PIN347 and ABCB19.

Historically, the role of basipetal auxin transport in shoot branching has been considered in the context of auxin exported from young expanding leaves at the primary shoot apex and carried down the stem, with auxin acting at each node to inhibit bud growth. According to this idea the absolute concentration of auxin at the node is read out to modulate bud activity, for example by regulating the synthesis of a second messenger that moves up into the bud. Strigolactone has been proposed to act in this way- as a second messenger for auxin [32]. In this scenario, low auxin transport in the stem should correlate with low auxin concentration at nodes and hence increased branching. This correlation is broken in strigolactone mutants, where high auxin transport and high stem auxin concentrations correlate with increased shoot branching [38]. That the auxin transport phenotype is directly associated with the branching phenotype is supported by the fact that treatment with low concentrations of auxin transport inhibitors such as NPA can restore wild-type branching levels in strigolactone mutants [38]. Here we extend this result, demonstrating that the *pin347* triple mutant reduces main stem auxin transport in strigolactone mutant backgrounds, and also reduces branching.

**Fig 9:**
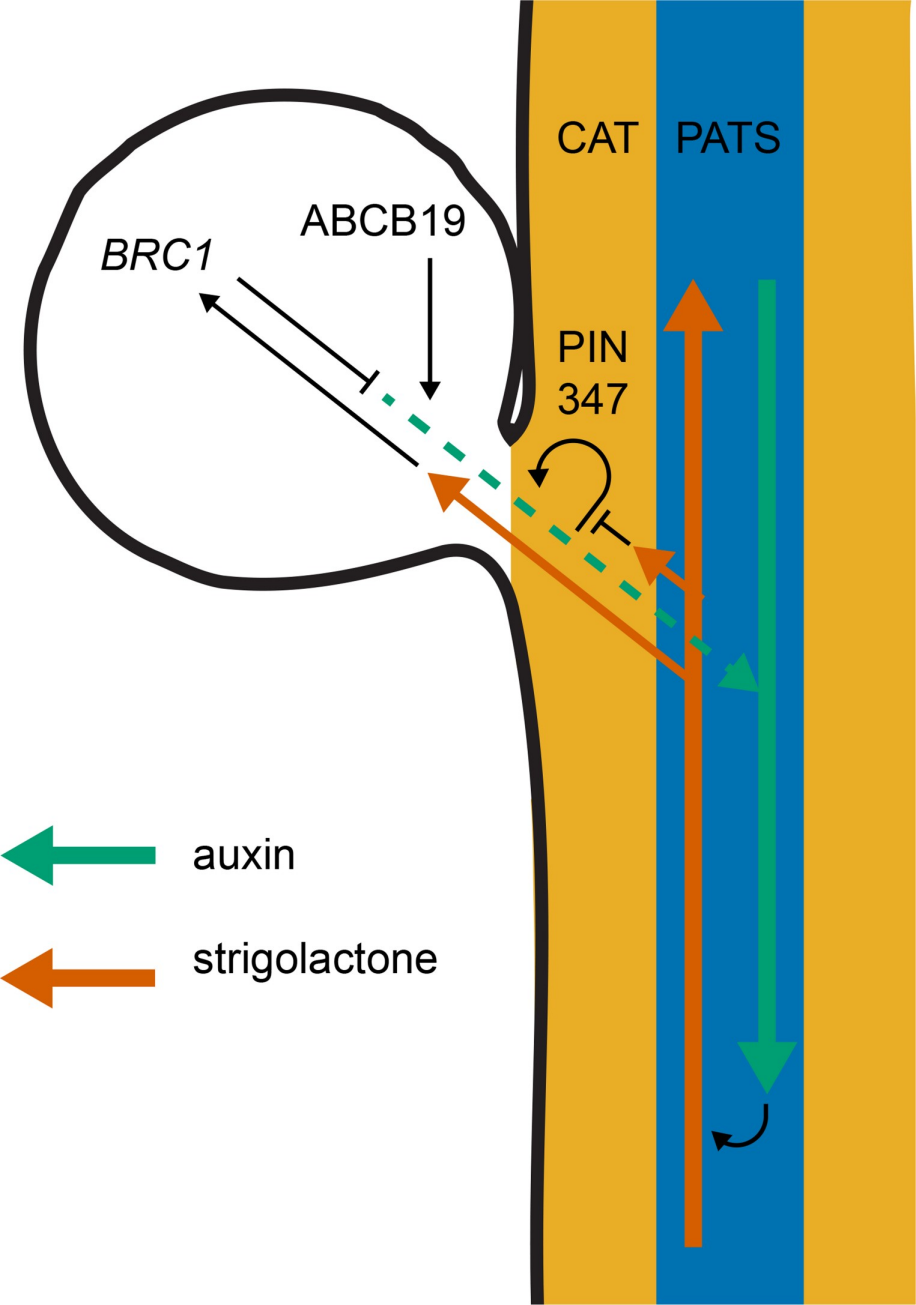
Model for bud activation. Cartoon of a nodal Arabidopsis stem segment with associated bud (leaf not shown). The coloured arrows indicate the flow of auxin (green) and strigolactone (red). The blue shading indicates the polar auxin transport stream (PATS), with PIN1 dominating, and the orange shading represents the connective auxin transport (CAT), with PIN3, PIN4 and PIN7 dominating. The black lines indicate hypotheses involving either promotion (arrowheads) or repression (end lines). Specifically, we have previously proposed that positive feedback between auxin flux and PIN polarisation drives canalisation of auxin transport between the bud and the main stem, and this is necessary for sustained bud activation. We have also previously proposed that strigolactone inhibits canalisation by triggering PIN1 endocytosis. Data presented here suggest that this might also be true for PIN7, but that the transport activities of PIN3, PIN4 and PIN7 are all important to allow maximal bud activation in strigolactone-defective mutants. Although it is possible that ABCB transporters are also important in the stem, the very different effects of the *abcb19* mutant compared to the *pin347* triple mutant lead us to propose that an important site of action is in the bud, where they may contribute to auxin loading, and therefore canalisation of auxin transport out of the bud. Consistent with this idea, the branchy phenotype of *brc1* mutants is partly suppressed in the *abcb19* mutant background. This is consistent with the idea that BRC1 acts at least in part through increasing bud auxin loading, either independently of upstream of ABCB19.

These data provide support for an auxin transport canalisation-based mechanism for bud activation, where bud activity does not depend straightforwardly on stem auxin concentration, but rather on the relative sink strength of the stem for auxin compared to the source strength of the bud, coupled with the degree of positive feedback between auxin flux out of the bud into the stem and auxin transporter accumulation and polarisation along this transport route. Furthermore, the data support the idea that strigolactone modulates shoot branching at least in part through effects on auxin transport canalisation between the bud and the main stem. PIN3 PIN4 and PIN7 accumulate in the tissues between the bud and the stem PATS and their mutation reduces branching in strigolactone mutants. This is not a generic effect, since there is no effect on branching in either the wild type, or the highly branched *brc1brc2* double mutant. This can be explained if strigolactone mutant buds activate because low PIN1 removal from the plasma membrane makes establishment of auxin transport canalisation between the bud and the stem easier to achieve. Under this model, reduced PIN347-mediated auxin flux from the bud across the CAT tissues and into the main stem PATS could work against the feedback in the canalisation process, restoring bud inhibition (Fig 9). The reduced bud activation rate of *pin347* mutants following decapitation is consistent with this idea. Similarly, the reduced ability of *pin347* mutant buds to communicate with each other in 2-node assays is consistent with reduced auxin flow across the stem.

This highlights an important feature of the 2-node assay. A low relative growth index can reflect two distinct system properties. Both buds could activate because each bud is difficult to inhibit, as in the case of strigolactone mutants, or both buds could activate because of their reduced ability to inhibit one another, as we propose for the *pin347* mutant. In this context it is interesting to note that *pin347* and *abcb19* explants behave similarly in 2-node assays. Both show reduced competition between buds and reduced strigolactone response compared to wild type. However, for a range of other branching phenotypes they show marked differences.

A particularly striking difference is that *abcb19* mutation reduces branching in both strigolactone and *brc1brc2* mutants, whereas *pin347* has no effect in a *brc1brc2* background. In addition, *abcb19* has a stronger effect on branching in wild-type plants than *pin347*. Another notable contrast is that of bud activation kinetics following decapitation, where *pin347* mutation slows activation rate late after decapitation, while *abcb19* mutation accelerates activation rate early after decapitation. Similarly, there are different effects on stem auxin transport dynamics, with evidence supporting a role for PIN347 in auxin exchange between the PATS and CAT, but no indication of a similar role for ABCB19, despite at least some effect of *abcb19* on bulk stem auxin transport. Consistent with these distinct modes of action are the additive effects of *pin347* and *abcb19* on branching in high branching environments and genetic backgrounds. Thus, PIN347 and ABCB19 have distinct roles in stem auxin transport and this is associated with distinct roles in bud activation (Fig 9).

Interpreting these results requires caution. According to the auxin transport canalisation-based model, bud activation depends on the relative auxin source strength of the bud, the sink strength of the stem and the degree of positive feedback between auxin flux and auxin transport in the direction of the flux. Changes in the auxin transport system can affect all of these properties simultaneously and in interconnected ways, making it difficult to predict the outcome of a simple intervention, or to establish straightforward causal relationships. These interconnections also mean that the same intervention can have opposite effects depending on the state of the system. For example, we have previously shown that strigolactone treatment can promote branching in genetic backgrounds with compromised PIN insertion, whereas it typically inhibits branching in wild-type backgrounds [22].

In the context of the genetic analyses we present here, it is tempting to speculate about the extent to which these different transporters act directly in the signalling pathways for the various players in the system. Interestingly, while PIN3 and PIN4 levels at the plasma membrane of xylem parenchyma cells are unchanged in the strigolactone mutants, PIN7 over-accumulates compared to wild type. This raises the possibility that PIN7, like PIN1, is a direct target for strigolactone signalling, whereas PIN3 and PIN4 are not. However, only the triple *pin347* mutant strongly suppresses the shoot branching phenotypes of strigolactone mutants. Therefore, as well as PIN7 potentially directly contributing to the strigolactone response, it is likely that expression of PIN347 in the stem tissue between the bud and the PATS is required for efficient auxin transport canalisation between the bud and the stem. This could result in strigolactone resistance and suppression of branching in strigolactone mutants by reducing positive feedback during auxin transport canalisation independently of the effect of strigolactone on this same process, acting via PIN7 and PIN1, in the same way that NPA treatment reduces branching in strigolactone mutants.

In this context it is interesting that *pin347* mutation does not affect *brc1brc2* branching. Current evidence suggests that strigolactone regulates bud growth in Arabidopsis by two separate mechanisms- inducing PIN1 endocytosis and up-regulating *BRC1* transcription. The mechanism of action of BRC1 in regulating bud growth is apparently independent of PIN1, and lack of suppression by *pin347* is consistent with this idea, while at the same time supporting the idea that *pin347* influences branching through effects on the positive feedback in canalisation.

In contrast *abcb19* mutation suppresses both strigolactone and *brc1brc2* mutants. The effect of *abcb19* could be a general bud suppressing effect, or it could be specifically downstream of strigolactone acting via *BRC1*. An attractive hypothesis is that ABCB19 is important for loading auxin into the bud PATS in the bud apex. This is consistent with its role in young seedlings, where auxin loading at the shoot apex has been shown to be deficient in *abcb19* mutants [45]. According to this model, *abcb19* mutants might have reduced bud auxin source strength as a result of poor auxin loading. This could account for the suppression of branching in *brc1brc2* by *abcb19*, particularly if BRC1 modulates bud activation potential by an effect on bud auxin source strength, either via ABCB19 or via a parallel mechanism (Fig 9). The strong reduction in ABCB19 accumulation in the *d14* mutant background is somewhat counterintuitive, since our double mutant analysis demonstrates that loss of *ABCB19* function suppresses branching in strigolactone mutants. On the other hand, this result does suggest a direct link between ABCB19 and strigolactone signalling which could be explained by negative feedback in the pathway. For example, as proposed above, strigolactone acting via *BRC1* may down-regulate ABCB19 activity, repressing shoot branching by reducing auxin loading in the bud. In strigolactone mutants ectopic bud activation could therefore be partly due to over-activation of ABCB19. If this hyperactivity triggers negative feedback in ABCB19 accumulation, then strigolactone mutants would both show reduced shoot branching and reduced ABCB19 accumulation.

Distinct effects of PIN347 and ABCB19 at different points in the canalisation process- auxin flux and therefore PIN1 accumulation, versus bud auxin loading- is consistent with the additive effects of their mutation on branching. The establishment of canalised auxin export, and therefore sustained bud growth, could be differently sensitive to changes in different parts of the system in different contexts. This could account for the different effects the auxin transporter mutations in different assays. For example, both *abcb19* mutation and *pin347* mutation suppress branching in strigolactone deficient mutants, but in decapitated wild-type plants, *abcb19* mutation accelerates bud activation, whereas *pin347* delays it, although for both the total number of buds activated eventually equilibrates at wild-type levels. In decapitated plants, sustained bud activation may be driven by high stem auxin sink strength. Under these circumstances, the positive feedback in flux-driven PIN accumulation along the route from the bud to the stem PATS might be important for rapid canalisation, whereas auxin loading in the bud may be less critical.

This explanation raises questions, which we hope to address in the future, about the importance of auxin loading at the bud apex for bud activation and bud-bud competition, and the role, if any, that *BRC1* plays in this process (Fig 9). Given the multiple levels of feedback in the shoot auxin transport network, further refinement of our computational models of auxin transport dynamics is likely to be essential in tackling these questions.

## Materials and methods

### Plant materials

*Arabidopsis thaliana* (‘Arabidopsis’) plants were all in the Col-0 ecotype. The *abcb1-100*, *abcb19-101* [46], *brc1-2brc2-1* [24], *d14-1* [37], *max2-1* [34], *max4-5* [38] and *pin3-3pin4-3pin7-1* [14], *PIN1*::*PIN1-GFP* [47], *PIN3*::*PIN3-GFP* [48], *PIN4*::*PIN4-GFP* [49], *PIN7*::*PIN7*:*GFP* [48] and *ABCB19*::*ABCB19-GFP* [50] lines have been described previously. The *abcb1-100*, *abcb19-101*, *pin3-3*, *pin4-3* and *pin7-1* alleles were verified using the primers and genotyping strategies described in [14]. The *brc1-2* and *brc2-1* alleles were verified using the primers and genotyping strategies in S1 and S2 Tables, respectively.

### Plant growth conditions

Plants were grown in controlled growth environment chambers (Conviron), set at 22/18°C day/night cycles. Light was supplied using white fluorescence tubes at an intensity of 170 μMm^-2^s^-1^ with 16/8 h (long days) or 8/16 h (short days) light/dark cycles. Plants were grown on Levington’s F2 compost (Levington Horticulture, Ipswich, UK) in P24 cellular trays (25 cm^2^ per pot).

### Phenotypic measurements

All measurements were conducted at terminal flowering, unless indicated otherwise. In our growth conditions this was approximately 7 weeks post-germination. Primary branching was quantified for each plant, counting the number of first-order cauline and rosette branches > 5 mm. Rosette branch activation was determined as previously described [14, 42], where the number of active rosette branches (> 5 mm) was determined daily post-decapitation. Branch angles were measured by photographing the junction of the primary inflorescence and the two most basal two cauline inflorescences. Using these images, the angle between the stem and the adaxial side of the branch was determined using Fiji. The branch angle of each plant was defined as the average of these two measurements. Stem diameter was determined using digital callipers (Mitutoyo 150 mm, 0.01 mm accuracy) by measuring the diameter of the primary inflorescence at the base. The widest and narrowest points were measured and the stem diameter of each plant was defined as the average of these two measurements.

### Hormone solutions

For strigolactone treatments GR24 (LeadGen Labs LLC) was dissolved in 90% acetone to a stock concentration of 50 mM. GR24 was used at a final concentration of 5 μM and for control treatments an equivalent amount of solvent was added.

### Physiological assays

*Arabidopsis thaliana* salts (ATS) was used for in vitro growth [51]. Plants were grown axenically in 1 litre Weck jars. Each jar contained 50 ml ATS with 1% sucrose and 0.8% agar. Hormones were added as described above. Plants were sown at a density of 7 plants per jar and kept in tissue culture growth conditions (16/8 h light/dark, 21/17°C day/night and 80 μMm^-2^s^-1^) for 8 weeks prior to scoring. Dry weight was determined at the end of the experiment for each plant by carefully removing the agar from the roots. Each plant was kept in a petri dish and dried in an oven at 50°C for 24 h. Samples were weighed using a laboratory balance scale (Sartorius CPA124S, d = 0.1 mg).

### Bud competition assay

Competition between buds was assayed by measuring growth of two cauline buds on an isolated 2-node stem segment, as described in [14]. Young inflorescences carrying three cauline buds, each less than 1 mm in length, were harvested and the shoot apex and most apical bud was removed under a stereo microscope, using the tip of a hypodermic needle. Samples were placed in Eppendorf tubes containing ATS solution and covered in parafilm. Bud length was measured using a ruler and the relative growth index was determined 10 days post decapitation, by calculating the proportion of branch length in the longest branch [39].

### Auxin transport assays

Standard bulk transport assays were performed as described in [14] with slight modifications. Stem segments, consisting of basal internodes 15 mm in length were collected and the apical ends were submerged in 30 μl ATS, containing 1 μM ^14^C-IAA (American Radiolabeled Chemicals) and 0.005% Triton X-100. Stem segments were left in solution for 6 or 18 hours, as described, and the basal 5 mm segment was excised, halved transversally and placed in 200 μl MicroScint-20 scintillation liquid (Perkin Elmer) and shaken for at least 6 hours at 400 RPM prior to scintillation counting.

Pulse assays were performed as described in [14]. Stem segments 24 mm in length were collected and the apical ends were submerged in 20 μl ATS containing 5 μM ^14^C-IAA and 0.005% Triton X-100 for 10 minutes. The samples were then transferred to fresh ATS solution without ^14^C-IAA and left for various time periods, as indicated. Samples were then cut into 2 mm segments and each segment was transferred to 200 μl scintillation liquid and shaken overnight at 400 RPM, prior to scintillation counting.

### Microscopy

Quantification of basal PIN localisation was carried out as described in [22]. The most basal internode of 6-week old plants carrying a functional *PIN1*::*PIN1-GFP* [47], *PIN3*::*PIN3-GFP* [48], *PIN4*:*PIN4-GFP* [49] or *PIN7*::*PIN7-GFP* [48] reporter construct were excised and longitudinal hand sections were made through the vascular bundles. These sections were covered in ATS solution and images were taken using a Zeiss LSM700 microscope fitted with a 20x water immersion objective. For GFP excitation the 488 nm laser intensity was typically 5–10% and kept constant within experiments. Background fluorescence and chloroplasts were obtained using the 639 nm laser at an intensity of 2–6%. Transmitted light images were recorded to verify tissue anatomy. Image analysis was carried out using Fiji and the mean fluorescence was determined by hand drawing a polygon across the relevant membrane, as described in [22]. Imaging of *ABCB19*::*ABCB19-GFP* was done in comparable tissue as those used for PIN GFP imaging but was conducted using a Leica SP8 FLIMan fitted with a 20x water immersion objective and laser intensities between 1–5%, which were kept constant between comparisons. Transmitted light images were recorded to verify tissue anatomy.

### Statistical analysis

All statistical tests were carried out using R, version 3.3.2. Unless indicated otherwise, linear models were fitted to the data. To check the underlying assumptions of the linear model, diagnostic plots for linear regression analysis were used. If the underlying assumptions were not violated the least-square means of the model were calculated using the *emmeans* package in R. Afterwards, Tukey’s HSD test was carried out and different letters were assigned to groups that were significantly different from each other at a threshold of *p* < 0.05. Where the assumptions of the linear model were violated, the data were analysed using a non-parametric approach. Pairwise Wilcoxon rank-sum tests were used and, where necessary, Holm-Bonferroni corrections were made.

For box plots, the box spans the first to third quartile and the line represents the median. The whiskers indicate the variability outside the upper and lower quartiles and outliers are indicated by individual points. Where used, the error bars indicate the 95% confidence interval, which was calculated as twice the standard error of the mean.

## Supporting information

S1 Table Genotyping primers. (DOCX)

S2 Table Genotyping strategies. (DOCX)

S1 Fig Shoot phenotypes of strigolactone mutants with impaired PIN347 function. (A) Mean number of active rosette branches over time following decapitation at day 0. Plants were grown under short day conditions for 4 weeks, shifted to long days to induce flowering and decapitated when the inflorescences reached 10 cm. The number of active rosette branches, defined as longer than 5 mm were counted daily. Error bars represent the 95% confidence interval of the mean. Non-overlapping error bars indicate statistical differences compared to *max2*, verified using non-parametric tests comparing wild type and each mutant to *max2* with a threshold of *p* < 0.05, with Holm-Bonferroni adjustment, *n* = 20–24. Branch angle (B, F), Plant height (C, G), Stem diameter (D, H) and Primary branches (E) at terminal flowering for the genotypes indicated. The boxes span the first to third quartile and the line represents the median. The whiskers indicate the variability outside the upper and lower quartiles and outliers are indicated by individual points. Tukey’s HSD tests were carried out after obtaining the least-square means for a linear model fitting the data. Different letters indicate statistically significant differences at *p* < 0.05, *n* = 12–20 for B-D and *n* = 20–24 for E-H.
(TIF)

S2 Fig Shoot phenotypes of strigolactone mutants with impaired ABCB function. Branch angle (A), Plant height (B), and Stem diameter (C) at terminal flowering for the genotypes indicated. The boxes span the first to third quartile and the line represents the median. The whiskers indicate the variability outside the upper and lower quartiles and outliers are indicated by individual points. Tukey’s HSD tests were carried out after obtaining the least-square means for a linear model fitting the data and different letters indicate statistically significant differences at *p* < 0.05. *n* = 20–24.
(TIF)

S3 Fig Shoot phenotypes of plants lacking PIN347 and ABCB19. Branch angle (A), Plant height (B), and Stem diameter (C) at terminal flowering for the genotypes indicated. The boxes span the first to third quartile and the line represents the median. The whiskers indicate the variability outside the upper and lower quartiles and outliers are indicated by individual points. Tukey’s HSD test were carried out after obtaining the least-square means for a linear model fitting the data and different letters indicate statistically significant differences at *p* < 0.05, *n* = 18–24.
(TIF)

S4 Fig *BRC1*-mediated branching is unaffected by ABCB1. Primary branch number at terminal flowering for plants of the genotypes indicated grown continuously under long day growth conditions (grey) or under short day conditions for four weeks and then shifted to long day conditions (magenta). The boxes span the first to third quartile and the line represents the median. The whiskers indicate the variability outside the upper and lower quartiles. The data for Col-0 and *brc1brc2* are the same as in Fig 7B. Tukey’s HSD test was carried out after obtaining the least-square means for a linear model fitting the data and different letters indicate statistically significant differences at *p* < 0.05, *n* = 20–24.
(TIF)
