# Supplementary material for: Connective auxin transport contributes to strigolactone-mediated shoot branching control independent of the transcription factor BRC1
Source: PLoS Genet. 2019 Mar 13;15(3):e1008023. doi: 10.1371/journal.pgen.1008023 (PMC6433298; doi:10.1371/journal.pgen.1008023)
Supplement: S1 Table — (DOCX) [file pgen.1008023.s001.docx]

| ID | Gene | Sequence | Direction |
| --- | --- | --- | --- |
| MVR228 | *brc1-2* | AACCAAACCATCCCAAAC | forward |
| MVR229 | *brc1-2* | ACCAAGTACCAATCCACA | reverse |
| MVR230 | *brc2-1* | CTTTTCTCCTCATCCACC | forward |
| MVR231 | *brc2-1* | CCTTCTTTTCCTTTCTCTTC | reverse |
| MVR036 | SALK LBb1.3 | ATTTTGCCGATTTCGGAAC | forward |
