# Supplementary material for: Connective auxin transport contributes to strigolactone-mediated shoot branching control independent of the transcription factor BRC1
Source: PLoS Genet. 2019 Mar 13;15(3):e1008023. doi: 10.1371/journal.pgen.1008023 (PMC6433298; doi:10.1371/journal.pgen.1008023)
Supplement: S2 Table — (DOCX) [file pgen.1008023.s002.docx]

| Gene | Wild type | Mutant allele |
| --- | --- | --- |
| *brc1-2* | MVR228 + MVR229 | MVR036 + MVR229 |
| *brc2-1* | MVR230 + MVR231 | MVR036 + MVR231 |
