# Supplementary figures and images for: Connective auxin transport contributes to strigolactone-mediated shoot branching control independent of the transcription factor BRC1
Source: PLoS Genet. 2019 Mar 13;15(3):e1008023. doi: 10.1371/journal.pgen.1008023 (PMC6433298; doi:10.1371/journal.pgen.1008023)

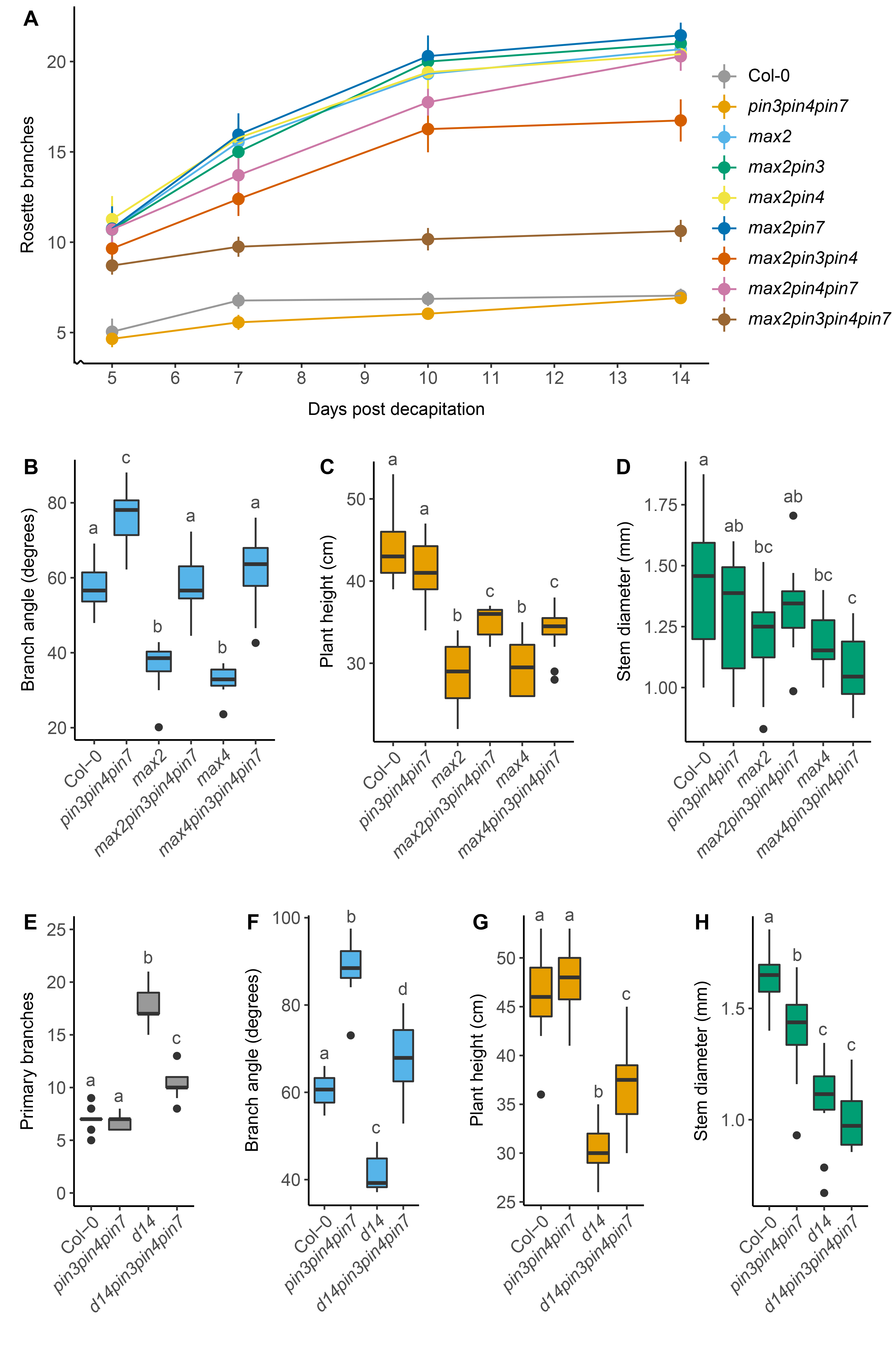

Supplement: S1 Fig — (A) Mean number of active rosette branches over time following decapitation at day 0. Plants were grown under short day conditions for 4 weeks, shifted to long days to induce flowering and decapitated when the inflorescences reached 10 cm. The number of active rosette branches, defined as longer than 5 mm were counted daily. Error bars represent the 95% confidence interval of the mean. Non-overlapping error bars indicate statistical differences compared to max2, verified using non-parametric tests comparing wild type and each mutant to max2 with a threshold of p < 0.05, with Holm-Bonferroni adjustment, n = 20–24. Branch angle (B, F), Plant height (C, G), Stem diameter (D, H) and Primary branches (E) at terminal flowering for the genotypes indicated. The boxes span the first to third quartile and the line represents the median. The whiskers indicate the variability outside the upper and lower quartiles and outliers are indicated by individual points. Tukey’s HSD tests were carried out after obtaining the least-square means for a linear model fitting the data. Different letters indicate statistically significant differences at p < 0.05, n = 12–20 for B-D and n = 20–24 for E-H. (TIF) [file pgen.1008023.s003.tif]

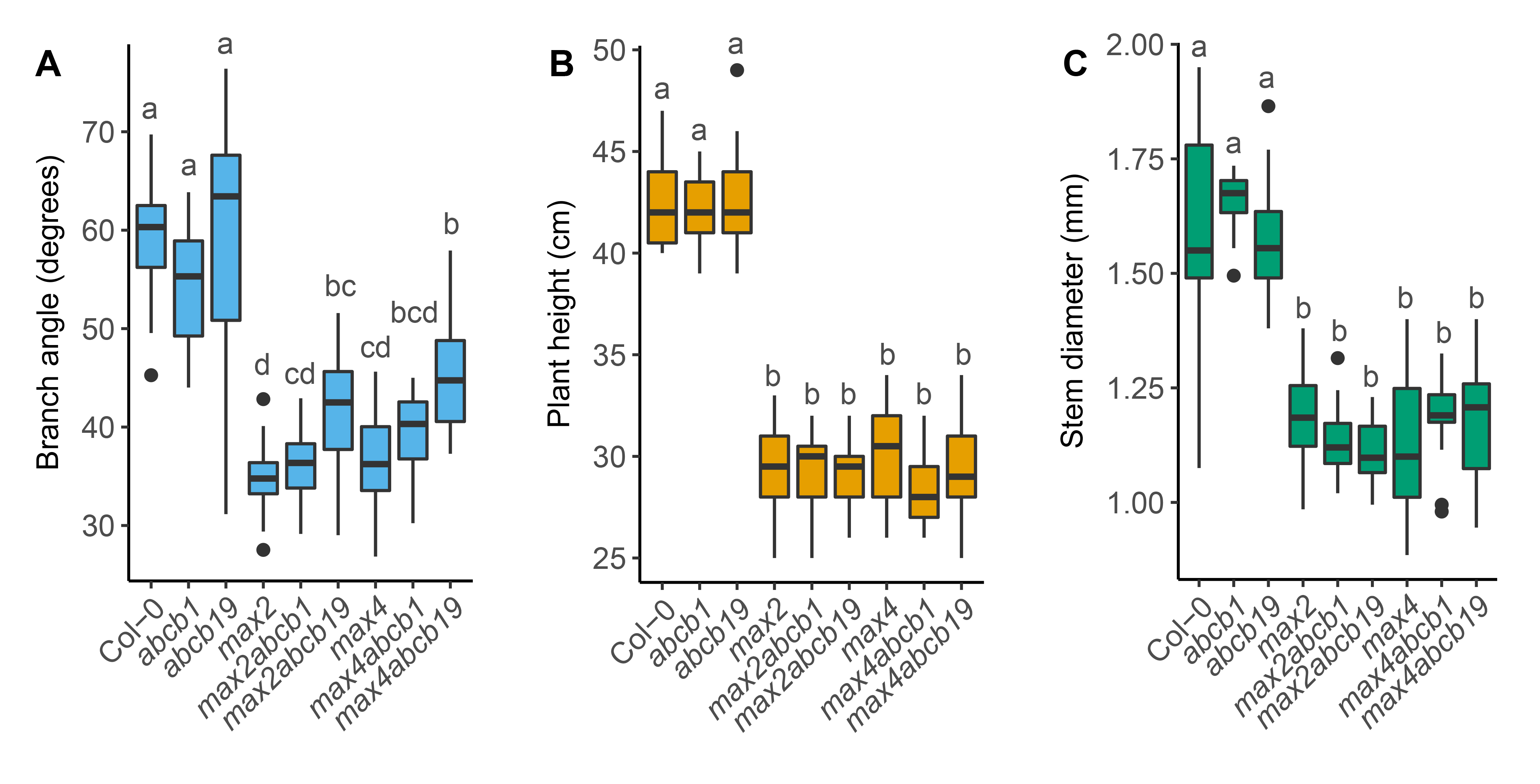

Supplement: S2 Fig — Branch angle (A), Plant height (B), and Stem diameter (C) at terminal flowering for the genotypes indicated. The boxes span the first to third quartile and the line represents the median. The whiskers indicate the variability outside the upper and lower quartiles and outliers are indicated by individual points. Tukey’s HSD tests were carried out after obtaining the least-square means for a linear model fitting the data and different letters indicate statistically significant differences at p < 0.05. n = 20–24. (TIF) [file pgen.1008023.s004.tif]

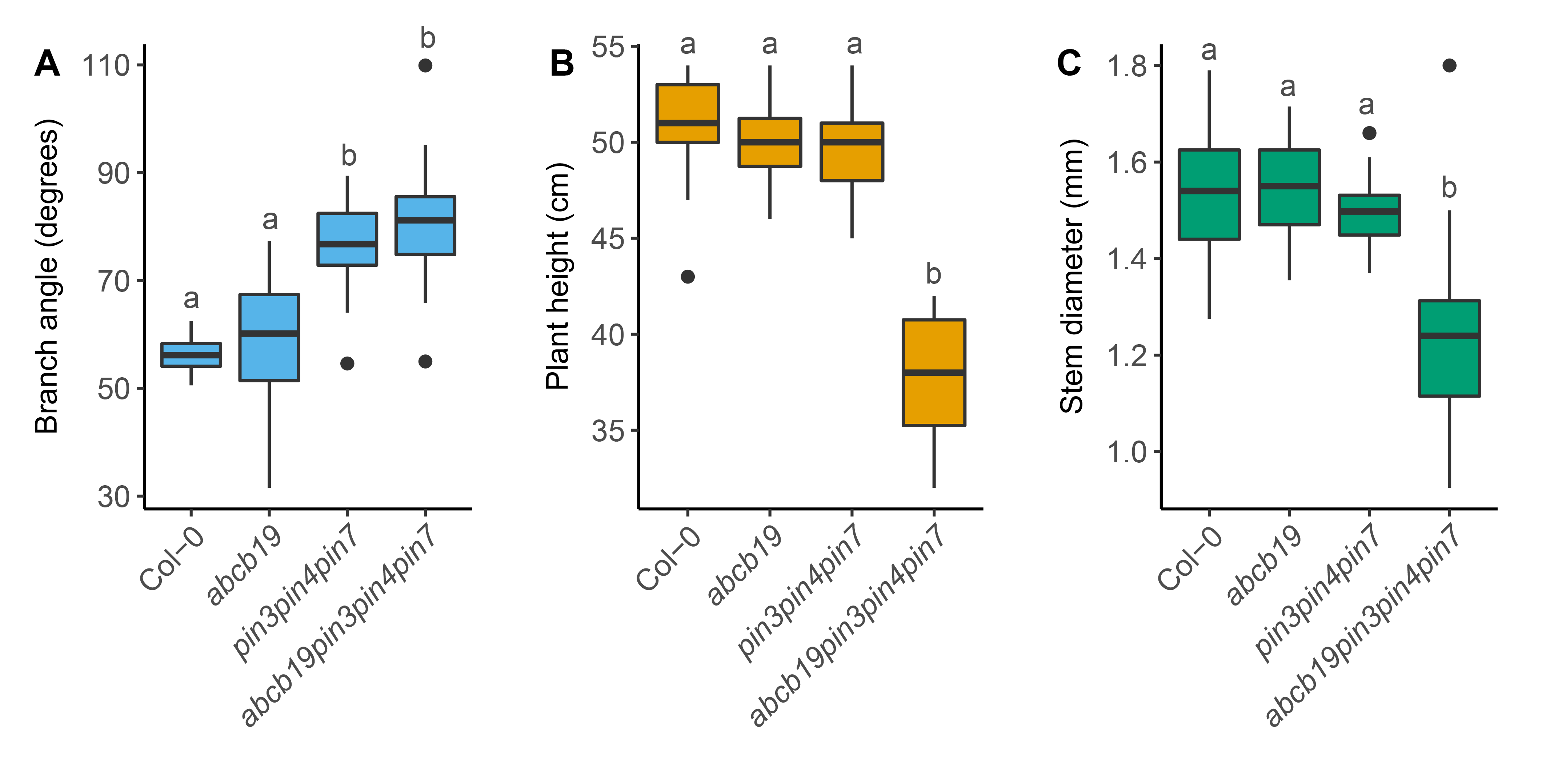

Supplement: S3 Fig — Branch angle (A), Plant height (B), and Stem diameter (C) at terminal flowering for the genotypes indicated. The boxes span the first to third quartile and the line represents the median. The whiskers indicate the variability outside the upper and lower quartiles and outliers are indicated by individual points. Tukey’s HSD test were carried out after obtaining the least-square means for a linear model fitting the data and different letters indicate statistically significant differences at p < 0.05, n = 18–24. (TIF) [file pgen.1008023.s005.tif]

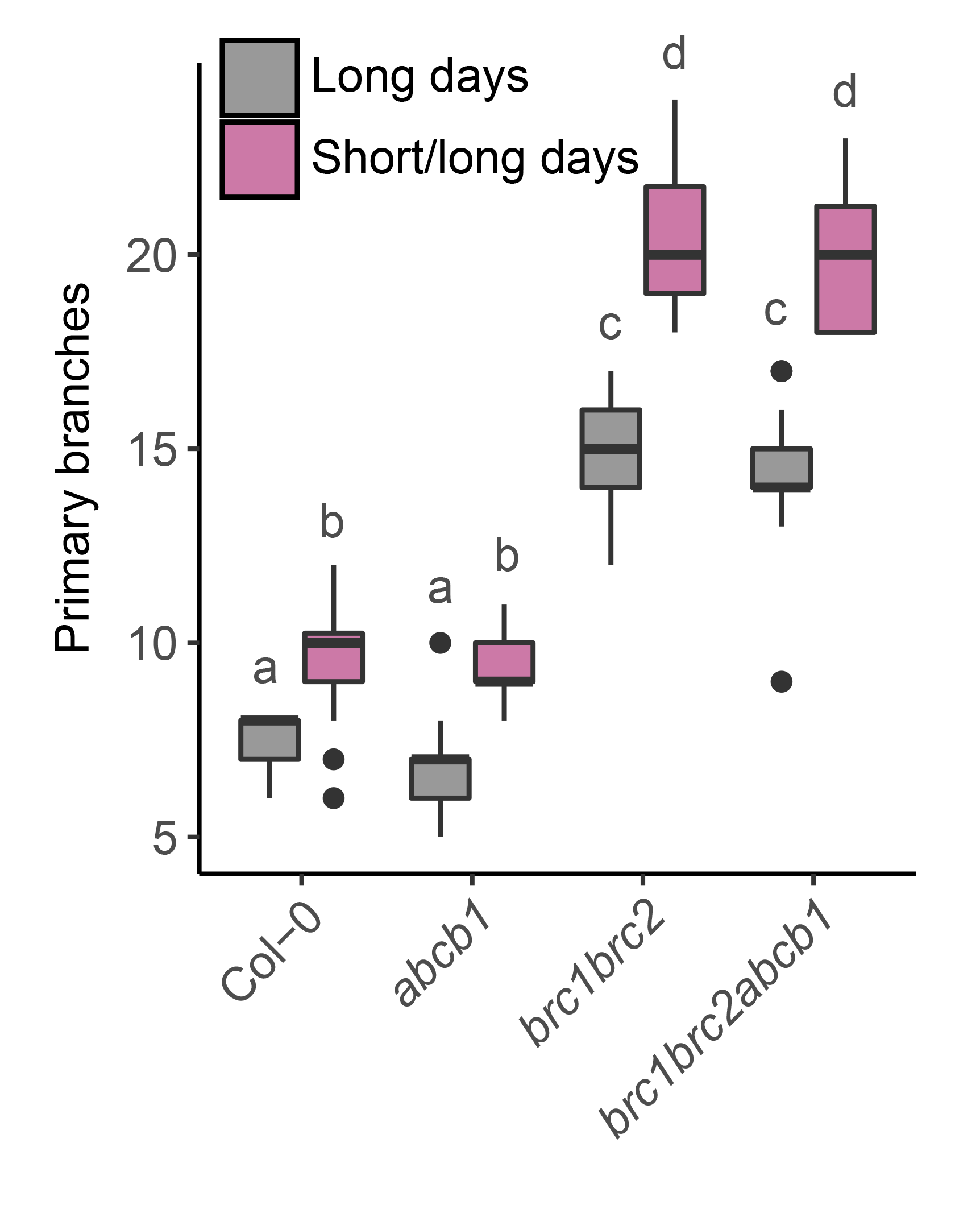

Supplement: S4 Fig — Primary branch number at terminal flowering for plants of the genotypes indicated grown continuously under long day growth conditions (grey) or under short day conditions for four weeks and then shifted to long day conditions (magenta). The boxes span the first to third quartile and the line represents the median. The whiskers indicate the variability outside the upper and lower quartiles. The data for Col-0 and brc1brc2 are the same as in Fig 7B. Tukey’s HSD test was carried out after obtaining the least-square means for a linear model fitting the data and different letters indicate statistically significant differences at p < 0.05, n = 20–24. (TIF) [file pgen.1008023.s006.tif]
